# Genomic and proteomic analysis of transcription factor TFII-I reveals insight into the response to cellular stress

**DOI:** 10.1093/nar/gku467

**Published:** 2014-05-28

**Authors:** Alex Xiucheng Fan, Giorgio L. Papadopoulos, Mir A. Hossain, I.-Ju Lin, Jianhong Hu, Tommy Ming Tang, Michael S. Kilberg, Rolf Renne, John Strouboulis, Jörg Bungert

**Affiliations:** 1 Department of Biochemistry and Molecular Biology, Center for Epigenetics, Genetics Institute, Powell Gene Therapy Center, Gainesville, Florida, USA; 2 Departmentof Biology, University of Crete, GR1409 Heraklion, Greece; 3 Divisionof Molecular Oncology, Biomedical Sciences Research Center “Alexander Fleming”, Vari GR 16672, Greece; 4 Departmentof Molecular Genetics and Microbiology, College of Medicine, University of Florida, Gainesville, Florida, 32610, USA

## Abstract

The ubiquitously expressed transcription factor TFII-I exerts both positive and negative effects on transcription. Using biotinylation tagging technology and high-throughput sequencing, we determined sites of chromatin interactions for TFII-I in the human erythroleukemia cell line K562. This analysis revealed that TFII-I binds upstream of the transcription start site of expressed genes, both upstream and downstream of the transcription start site of repressed genes, and downstream of RNA polymerase II peaks at the ATF3 and other stress responsive genes. At the ATF3 gene, TFII-I binds immediately downstream of a Pol II peak located 5 kb upstream of exon 1. Induction of ATF3 expression increases transcription throughout the ATF3 gene locus which requires TFII-I and correlates with increased association of Pol II and Elongin A. Pull-down assays demonstrated that TFII-I interacts with Elongin A. Partial depletion of TFII-I expression caused a reduction in the association of Elongin A with and transcription of the DNMT1 and EFR3A genes without a decrease in Pol II recruitment. The data reveal different interaction patterns of TFII-I at active, repressed, or inducible genes, identify novel TFII-I interacting proteins, implicate TFII-I in the regulation of transcription elongation and provide insight into the role of TFII-I during the response to cellular stress.

## INTRODUCTION

TFII-I, also known as general transcription factor 2 I (GTF2I), belongs to a family of related proteins that act as transcription regulators (1,2). Genes encoding members of this transcription factor (TF) family are deleted in Williams–Beuren Syndrome patients (3). Mice homozygous for a deletion of the TFII-I gene die early during embryonic development with multiple defects (4). TFII-I is an unusual TF; it is relatively large containing multiple protein–protein interaction domains, and it associates with both expressed and repressed genes (1,2,5). Functional studies have shown that TFII-I can act as activator or repressor of transcription, which is likely determined by the sequence context and/or the interacting co-regulatory proteins.

TFII-I contains six R-repeats, also called I-domains, that resemble but are different from the helix-loop-helix domain (6). In addition, it also harbors a leucine zipper at the N-terminus as well as a basic region involved in DNA binding and a nuclear localization sequence (NLS). TFII-I is subject to post-transcriptional regulation by alternative splicing and phosphorylation (3,7–9). Alternative splicing generates isoforms that are expressed at different levels in different tissues (1,2). Phosphorylation of TFII-I has been shown to regulate its translocation from the cytoplasm to the nucleus (10,11). TFII-I is one of a few TFs for which specific functions in the cytoplasm have been demonstrated (1,2). For example, TFII-I has been shown to control the nuclear translocation of c-Rel, a regulator of the c-Myc TF (12). Furthermore, TFII-I has been shown to associate with phospholipase C-γ (PLC-γ) and to compete for interactions between PLC-γ and the transient receptor potential cation channel subfamily C member 3 (TRPC-3), thereby inhibiting agonist-induced calcium entry (13).

Originally, TFII-I was isolated and characterized as an activity that binds to the initiator sequence and mediates transcription of genes that contain this basal promoter element (14). *In vitro* studies demonstrated that TFII-I interacts with the TFII-D complex and that it is able to recruit transcription complexes to TATA-less promoters. While binding of TFII-I to the initiator has been demonstrated for a number of genes, TFII-I is not considered a general TF but rather as a protein that regulates transcription of a selected number of genes (1,2). In addition to interacting with the initiator, TFII-I has been shown to interact with E-box elements, mediated through interactions with TFs including the bHLH proteins, USF and c-Myc (15,16). Furthermore, TFII-I interacts with the so-called DICE (Downstream Immunoglobulin Control Element) sequences originally identified in specific immunoglobulin genes (17).

TFII-I has been reported to regulate many different genes that encode proteins involved in cell-cycle regulation, TGFβ-signaling, endoplasmic reticulum stress response and immune signaling (1,2). Moreover, TFII-I regulates genes that are expressed in a cell-type specific manner including the vascular endothelial growth factor receptor-2 and the β-globin genes (18,19). TFII-I activates and represses transcription by recruiting either co-activators or co-repressors, including histone deacetylases, to target genes (2,20,21).

A recent genome-wide ChIP/chip analysis of TFII-I in embryonic stem cells and in embryonic tissues revealed that TFII-I binds to a large number of genes involved in diverse cellular processes (5,22). The correlation between binding and gene expression was found to be low. This could be consistent with its dual function as an activator or repressor. However, Bayarsaihan *et al.* presented evidence that TFII-I interacts with poised genes in embryonic stem (ES) cells that are characterized by bivalent histone modifications, meaning the genes are associated with nucleosomes that carry both the H3K4me3 modification, typically found in transcribed genes, and the H3K27me3 modification, usually associated with repressed genes (5,22). The authors speculated that TFII-I may be involved in the marking of genes that are expressed during lineage commitment steps. A switch from repressor to activator function of TFII-I has been reported to be involved in immunoglobulin heavy chain (IgH) gene transcription (23). The repressed IgH-gene is associated with TFII-I and HDAC3. Upon activation, transcription co-factor OCA-B replaces HDAC3 from TFII-I and mediates interactions between the IgH promoter and an enhancer element.

In the present study, we identified TFII-I interacting proteins and determined sites of chromosomal binding of TFII-I in human K562 erythroleukemia cells. We generated and expanded single K562 cell clones expressing the *Escherichia coli* BirA ligase, which catalyzes biotinylation, or the *E. coli* BirA ligase in addition to TFII-I fused to a biotinylatable tag (24). Cells expressing BirA, or BirA together with biotinylatable TFII-I were subjected to high-throughput sequencing and protein interaction analysis. The data reveal novel interactions between TFII-I and nuclear proteins involved in transcription regulation and identify DNA elements associated with TFII-I in erythroleukemia cells.

## MATERIALS AND METHODS

### DNA constructs, cell culture and stable transfections

For N-terminal biotin tagging of TFII-I, the coding sequence of TFII-I was PCR amplified from plasmid pTO-TFII-I (19) using the following primers: Fwd, 5′-GCCGGCGGCCGCCCATATGGCCCAAGTTGC-3′ and Rev, 5′-CTGATCAGCGGGTTTAAACGGG3–3′. Restriction enzyme sites for *Not*I and *Pme*I were incorporated at the 5′ and the 3′ end, respectively, by the PCR reaction. The vector AviTEVFLAG/3xHANLSBirA/pBUDNeo was digested with *Not*I and *Pme*I, treated with calf intestine phosphatase and ligated with gel purified TFII-I coding fragment to generate pFlag-biotin-TFII-I. The entire coding sequences of TFII-I and BirA were verified by DNA sequencing.

K562 cells were grown in RPMI 1640 (Corning, Cellgro) containing 10% fetal bovine serum (FBS) and 1% penicillin-streptomycin. The cells were grown in 5% CO_2_ at 37°C and kept at a density between 1 × 10^5^ and 2 × 10^6^ cells/ml. Transfection of the pFlag-biotin-TFII-I expression construct or of the control vector expressing only BirA was carried out by nucleofection using the Amaxa Nucleofector kit (Amaxa, VCO-1001N) according to the protocol provided by the manufacturer. At 40 h after transfection, cells were subjected to G418 selection at 350 or 400 μg/ml for 14 days before cells were frozen down or expanded and subjected to western blotting experiments, protein purification or ChIP analysis. K562 cells expressing the Flag/biotin-tagged TFII-I were subjected to single clone selection using dilution and growth in 96-well plates. Single clones were expanded and subjected to western blot analysis using Flag-specific antibodies.

### Streptavidin-mediated protein pull-down

Nuclear extracts were prepared from K562 cells expressing BirA or BirA together with bio-tagged TFII-I according to a procedure published previously (25). Streptavidin-mediated pull-down was performed as described by Rodriguez *et al.* (26,27) with minor modifications. Briefly, 2 mg nuclear extract was used for each pull-down using 50 μl/mg streptavidin-coated magnetic beads (Dynabeads M-280, Life Technologies AS., Oslo, Norway). The beads were washed 3 times with phosphate buffered saline (PBS) at room temperature (RT) and then blocked with 200 μg/ml chicken egg albumin (CEA, Sigma Alderich, A5503-IG) in a final volume of 1 ml in HENG buffer (10 mM HEPES [pH 9], 1.5 mM MgCl_2_, 0.25 mM EDTA, 20% glycerol, 1 mM PMSF, 1mM DTT) while rotating at RT for 1 h. The salt concentration of the nuclear extract was adjusted to 150 mM NaCl and protease inhibitors (Complete, Roche) and NP-40 (0.3% final concentration) were added. After removing the blocking buffer, the beads were mixed with the nuclear extract in HENG buffer (containing 150 mM NaCl, 0.3% NP-40, protease inhibitors) and incubated in the presence of 5 μl/mg protein benzonase (Novagen, 25 U/μl) for 2 h to overnight at 4°C while rotating. The beads were then concentrated on a magnetic rack and washed 6 times for 10 min using HENG buffer with 300 mM NaCl and 0.3% NP-40. The beads were resuspended in 1 × Laemmli buffer and the proteins were eluted by boiling. Proteins were subjected to sodium dodecyl sulphate-polyacrylamide gel electrophoresis (SDS-PAGE) followed by western blotting or trypsin digestion of gel pieces. The trypsin-digested samples were then analyzed using an LTQ Orbitrap XL mass spectrometer system (Thermo Scientific, Waltham, MA, USA) and analyzed as described previously (28).

### Streptavidin-mediated pull-down of chromatin and massive parallel sequencing

Streptavidin-mediated pull-down of cross-linked biotinylated chromatin was performed by the procedure published by He and Pu (29). Briefly, 4 × 10^7^ K562 cells expressing either BirA or BirA together with bio-tagged TFII-I (clones 5 and 18) were cross-linked with 1% formaldehyde for 7 min. The reaction was quenched by adding glycine to a final concentration of 125 mM. After a wash in hypotonic buffer, the cross-linked material was resuspended in ChIP dilution buffer and subjected to sonication to yield fragments with an average length of 150–200 bp. An aliquot of 25 μl was taken and stored at −20°C. 50 μl Dynabeads M-280 Streptavidin (Life Technologies AS., Oslo, Norway, 11205D) and Dynabeads Protein A (Life Technologies AS., Oslo, Norway, 10002B) (per 6 × 10^7^ cells) were washed three times in PBS/1% bovine serum albumin and blocked for 1 h at 4°C. Sheared chromatin was pre-cleared using Protein A beads at 4°C for 1 h. The protein A beads were centrifuged and the supernatant was incubated together with the magnetic beads at 4°C overnight. The bound material was collected with a magnet and the beads were washed several times using SDS wash buffer, high-salt buffer, LiCl buffer and finally Tris-EDTA (TE) buffer as outlined by He and Pu (29). The cross-links, including that of the input samples, were reversed by incubating the beads in SDS-ChIP dilution buffer and incubation at 70°C overnight. The samples were treated with proteinase K and RNAse and DNA was purified using the QIAquick PCR purification kit as per the manufacturer's instruction. The quality of the bioChIP DNA was verified by qPCR using c-Fos promoter primers and examined using the Agilent 2100 Bioanalyzer (Agilent, Santa Clara, CA, USA). The bioChIP-seq DNA was then converted to a library for sequencing on the Illumina Genome Analyzer 2 according to the Illumina Multiplexing Sample Preparation Guide (www.illumina.com). Deep sequencing was carried out for two independent clones of bioTFII-I (clones 5 and 18) and one clone of BirA transfected K562 cells. All 36-nucleotide sequence reads produced were mapped to the UCSC hg18 Human Genome Assembly using the bowtie.2 algorithm (30). Sequence reads with multiple genome alignments and/or more than 2 nucleotide mismatches were excluded from subsequent analysis, whereas identical sequence reads were counted as one. Peak calling was performed using the Model-based Analysis for ChIP-Seq (MACS) algorithm (31) with default parameters. The two bioTFII-I aligned reads were merged together and analyzed versus the BirA data set using default parameters. Gene mapping was performed in the R environment using the ChIPpeakAnno package (32) and the TSS.human.NCBI36 gene location reference.

Peak locations for all K562 data sets used in this study were directly downloaded from the Encode database (http://genome.ucsc.edu/ENCODE/) (33,34). Accession numbers of the data sets used here are included in Supplementary Table S1. Overlaps were computed using the ‘find-OverlappingPeaks’ function included in the ChIPpeakAnno package (32).

### Co-immunoprecipitation (Co-IP) and immunoblotting

Co-IP was carried out as described by Rodriguez *et al.* (26). Briefly, 50 μl magnetic protein G beads (Dynabeads Protein G, Life Technologies AS., Oslo, Norway) per 500 μg protein were rinsed three times with PBS and twice with 100 mM sodium citrate (pH 5.0). Antibodies (6–8 μg per 500 μg protein) were added to the beads in 1 ml HENG buffer (10 mM HEPES-KOH, pH 9.0, 1.5 mM MgCl2, 0.25 mM EDTA, 20% glycerol, 1 mM PMSF and 1 mM DTT) and the samples were incubated for 2 h at RT while rotating. The beads were rinsed twice with 100 mM sodium citrate (pH 5.0) and once with 200 mM triethanolamine (pH 8.2). After addition of 20 mM dimethyl pimelimidate dihydrochloride (Sigma-Alderich, D8388, in 200 mM triethanolamine, pH8.2) the beads were incubated for 45 min at RT while rotating. The beads were rinsed once with 50 mM Tris, pH 7.5, and three times with PBS containing 0.01% Tween 20. The immunoglobulin G (IgG) and antibody beads were blocked with 200 μg/ml CEA by incubating while rotating for 1 h at RT. Nuclear protein extracts were incubated for 30 min at RT with 2.5 μl benzonase (Novagen) per 500 μg protein and then diluted with HENG buffer to bring the KCl concentration to 125 mM. The nuclear extracts were pre-cleared with the blocked IgG beads for 1 h at 4°C, and then transferred to the antibody beads and incubated overnight at 4°C. The IgG and control beads were washed 5 times for 5 min with HENG wash buffer (HENG buffer plus 300 mM KCl) at 4°C, rinsed twice with PBS and proteins were eluted off the beads by incubation in 30 μl 1xLaemmli buffer for 10 min at 80°C. The immunoblotting procedure was performed as described by Barrow *et al.* (35). Briefly, 10–20 μg protein was loaded onto 4–15% (wt/vol) TGX Tris-HCl gels (Bio-Rad) and subjected to electrophoresis. The proteins were transferred to Polyvinylidene fluoride (PVDF) membranes and incubated with antibodies. The following antibodies were used in these experiments: from Santa Cruz Biotechnology, αTFII-I (sc-9943), αBrg1 (sc-374197), αTopoIIα (sc-5346), αGAPDH (sc-25778), rabbit αIgG (sc-2027), mouse αIgG (sc-2025); from Bethyl Laboratories, αSMARCC2 (A301-038A), αTopoIIβ (A300-950A), αTCEB3 (A300-942A); from Cell Signalling, αSMARCC1 (11956S); from Biolegend, αTAF15 (ab-134616); from Affinity Bioreagents α-HA (OPA1-10980); from Sigma, α-FLAG M2 (F3165).

### RNA interference

The TFII-I target siRNA as well as the negative control siRNA pools and the transfection reagent were purchased from Thermo Scientific (siGENOME GTF2I SMARTpool [M-013638-00-0005], siGENOME Non-Targeting siRNA #2 [D-001210-02-05] and DharmaFECT1 [T-2001-03]). siRNA-mediated knockdown was performed in K562 cells according to the siRNA transfection protocol provided by Thermo Scientific DharmaFECT. Briefly, the siRNA pools and DharmaFECT transfection reagent were diluted in serum-free RPMI medium and incubated for 5 min at RT. Then the siRNA pools and DharmaFECT transfection reagent were mixed together and incubated at RT for 20 min. Antibiotic-free complete RPMI medium and the ready-to-use siRNA transfection mix was added to K562 cells using two concentrations of the TFII-I siRNA pool (25 and 50 nM) in separate experiments. Then, the cells were incubated at 37ºC in 5% CO_2_ for 24–48 h for mRNA analysis or 48–96 h for protein analysis.

For generating stable K562 cells expressing TFII-I specific (shTFII-I) or scrambled shRNA (scrambled control, SC) cells were transfected with the pGIPZ-shTFII-I or the pGIPZ-shSc vectors (Thermo Scientific) using lipofectamin 2000 (Invitrogen) according to the procedure provided by the manufacturer. Briefly, 5 × 10^5^ cells were transfected with 5 μg DNA in 2 ml of RPMI medium supplemented with 10% FBS and 1% penicillin/streptomycin. Cells were subjected to selection in the presence of 2 μg/μl puromycin. Subsequently, single cell clones were selected and the transfected K562 cells were maintained and expanded in RPMI medium containing 10% FBS, 1% penicillin/streptomycin and 1 μg/μl puromycin. The following antisense sequences were used: shRNA TFII-I, 5′-TTCATACACTGCAATGCAG-3′; SC, 5′-TCTCGCTTGGGCGAGAGTAAG-3′.

### RNA extraction, cDNA synthesis and quantitative PCR

RNA was extracted from wild-type K562 cells or K562 cells expressing BirA or BirA together with bio-tagged TFII-I (clones 5 and 18) using the RNeasy kit (Qiagen) and reverse transcribed using the Iscript cDNA synthesis kit (Bio-Rad). Quantitative PCR was performed as described before (35) using primers listed in Supplementary Table S2.

### Chromatin immunoprecipitation

ChIP was performed as described by Barrow *et al.* (35) with minor modifications. Briefly, 2 × 10^7^ WT K562 cells were cross-linked in 2% formaldehyde for 10 min at RT. After quenching the reaction with glycine at 125 mM, the chromatin was sonicated to yield fragments of 100–600 bp. For pre-clearing, the lysates with the cross-linked chromatin fragments were incubated with IgG (Santa Cruz; sc-2025) for 2 h at 4°C and then incubated with Dynabeads Protein A/G (Life Technologies AS., Oslo, Norway, 10002B/D). The lysates with the protein A beads were put on a magnetic rack and the supernatant was incubated with specific antibody on a rotating wheel at 4°C overnight. The beads were then washed with a series of several low-salt, high-salt, and LiCl buffers. After reversal of the crosslink at 65°C overnight, the DNA was purified by phenol/chloroform/isoamylalcohol and chloroform extractions and precipitated with 2.5 x volume 100% ethanol. The pellets were washed with 70% ethanol, resuspended in 10 mM Tris-Cl (pH 8.5), and analyzed by qPCR as described previously (35). The antibodies used for the ChIP are described under the section Co-IP and western blotting. Primer sequences for the qPCR analysis are listed in Supplementary Table S2.

## RESULTS

### Generation of K562 cells expressing biotinylated TFII-I

To identify TFII-I target genes and interacting proteins, we generated human erythroleukemia (K562) cells expressing the delta isoform of TFII-I bearing a biotinylatable tag (bioTFII-I). Protein-biotinylation allows for highly efficient and specific pull-down and isolation of protein complexes and protein/chromatin fragments using streptavidin beads (24,26–28). Furthermore the biotinylation approach allowed us to focus on one specific isoform of TFII-I. There are four different isoforms of TFII-I (1,2). The TFII-I delta isoform is ubiquitously expressed and we therefore decided to tag this isoform. Previous studies have analyzed TFII-I genome-wide occupancy in differentiating ES cells using ChIP followed by hybridization to microarrays (ChIP on Chip) (5,22). The authors used antibodies against TFII-I which did not discriminate between the different TFII-I isoforms (5,22).

**Figure 1.**
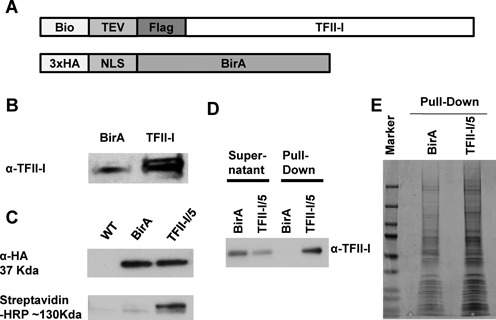
Identification of TFII-I interacting proteins in K562 cells by streptavidin-mediated pull-down and mass spectrometry analysis. (A) Structure of bio-tagged TFII-I and HA-tagged BirA. A cDNA construct was generated expressing TFII-I with an N-terminal tag consisting of the biotinylatable peptide (Bio), a TEV cleavage site and a Flag-peptide, as well as the *E. coli* BirA protein biotin ligase containing an N-terminal HA tag (3xHA) and a nuclear localization signal (NLS). (B) Nuclear extracts from K562 cells expressing BirA (BirA) or BirA together with the bio-tagged TFII-I were fractionated by SDS-PAGE and analyzed by western blotting with antibodies specific for TFII-I (α-TFII-I). (C) Nuclear extracts from K562 cells (WT), K562 cells expressing BirA or single cell clone 5 expressing bio-tagged TFII-I and BirA (TFII-I/5), were fractionated by SDS-PAGE, transferred to a PVDF membrane and examined using an HA-specific antibody (α-HA, top) or streptavidin conjugated horse radish peroxidase (Streptavidin-HRP, bottom). (D) Single K562 cell clone 5 expressing bio-tagged TFII-I and BirA (TFII-I/5), or cells expressing only BirA were subjected to pull-down using streptavidin-coated magnetic beads. The supernatant and pull-down material was subjected to western blotting using antibodies specific for TFII-I (α-TFII-I) (E) Representative image of coomassie-blue stained SDS-PAGE gels loaded with streptavidin pulled-down material from K562 cells expressing only BirA (BirA) or BirA together with bio-tagged TFII-I, in this case representing clone 5.

We used a DNA plasmid that contained coding fragments for a biotinylatable tag, a Flag-tag and a TEV protease cleavage site (Figure 1A). The insertion of TFII-I cDNA into this plasmid generated a coding sequence that, when expressed, introduces the Flag/bio-tag into the N-terminus of TFII-I. The plasmid also contains the cDNA for the *E. coli* BirA protein ligase, which catalyzes the addition of biotin to the biotinylatable tag of the recombinant TFII-I (36,37). Plasmids expressing the bio-tagged TFII-I and BirA (pFlag-biotin-TFII-I) or BirA only were transfected into K562 cells by nucleofection. Nuclear extracts from stably transfected cells expressing bio-tagged TFII-I/BirA or BirA alone were subjected to western blotting, and antibodies specific for TFII-I marked a novel band in the cells expressing BirA and the tagged protein that migrated slightly slower than TFII-I, due to the inclusion of the tag (Figure 1B). We generated two single cell clones that express biotinylated TFII-I at equivalent levels (clone 5 and 18). HA-tagged BirA is expressed only in cells expressing BirA or in cells expressing BirA and the bio-tagged TFII-I (clone 5, Figure 1C, top). Biotinylated TFII-I is only detectable in cells expressing both BirA and the tagged TFII-I (Figure 1C, bottom). We next prepared nuclear protein extracts from cells expressing BirA only or cells expressing BirA and biotin-tagged TFII-I (clone 5). These extracts were subjected to pull-down using streptavidin-coated magnetic beads. Both the pull-down material as well as the supernatant was subjected to SDS-PAGE and western blotting using antibodies specific for TFII-I (Figure 1D). The data show that TFII-I is efficiently pulled-down from extracts containing the biotinylated TFII-I but not from extracts that harbor the BirA ligase only. Figure 1E shows a coomassie-blue stained SDS-PAGE gel with pulled-down material from cells expressing BirA or cells expressing both BirA and the bio-tagged TFII-I. The summary data demonstrate that the tagged TFII-I was expressed in the K562 cells and that it was biotinylated.

### Genome-wide binding pattern of TFII-I in K562 cells

**Figure 2.**
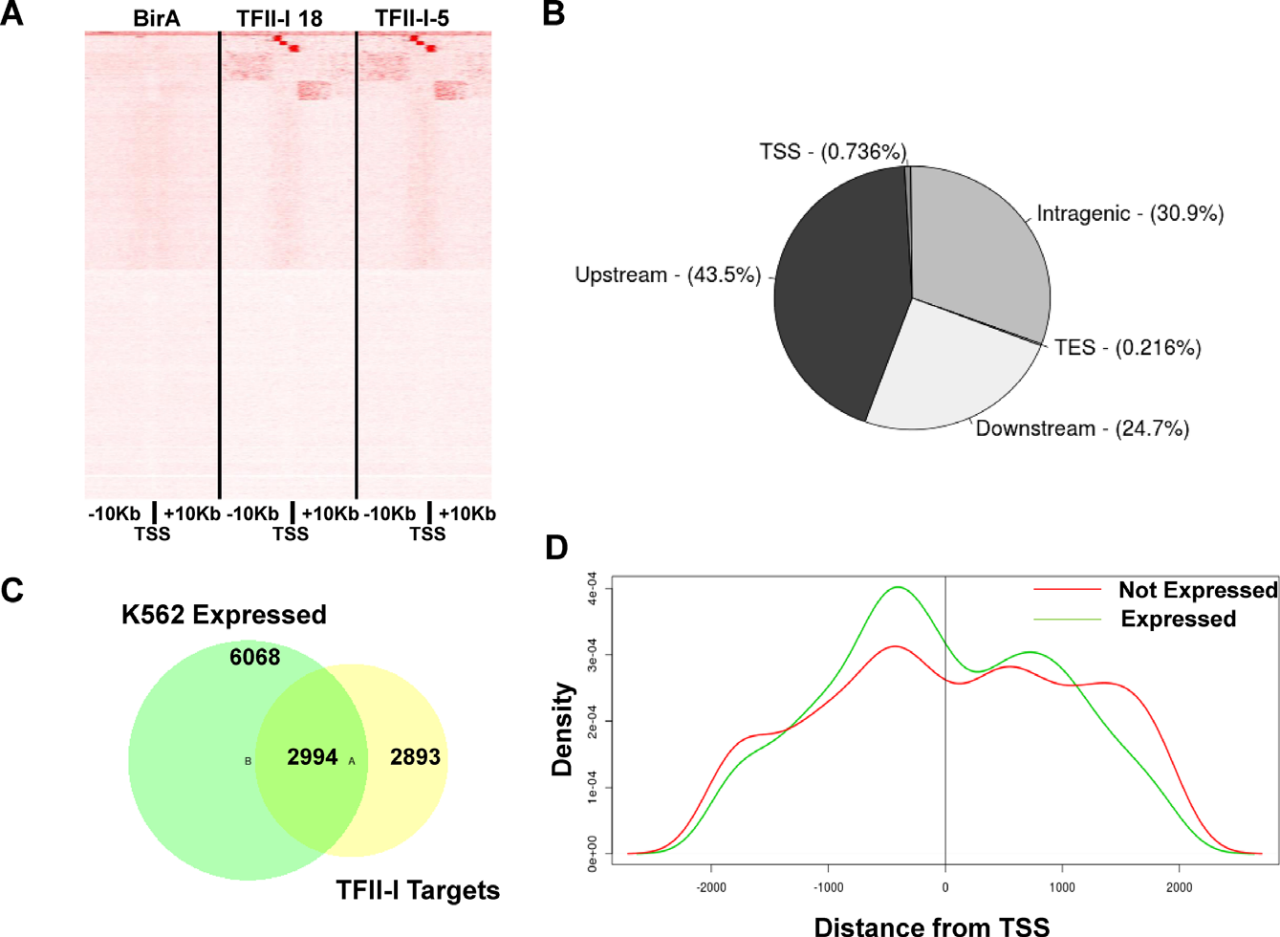
Features of global TFII-I genomic interactions. (A) Distribution of raw sequencing reads 10 kb upstream and downstream of all annotated TSS in cells expressing BirA only, or BirA together with biotinylated TFII-I (clones 5 and 18 as indicated). (B) Distribution of TFII-I binding peaks with respect to the location of genes. TSS intragenic (exons or introns), TES, Downstream (downstream of the coding region), Upstream (upstream of the coding region). (C) Correlation of TFII-I binding peaks and gene expression in K562 cells. Out of 6068 expressed genes in K562 cells, 2994 reveal a peak for TFII-I binding. (D) Distribution of TFII-I binding peaks with respect to the TSS in expressed (green) and silent (red genes) genes in a 2 kb window. The 0 depicts the TSS, – denotes upstream of the TSS and + denotes downstream of the TSS.

To analyze the global association of TFII-I with chromatin in K562 cells we performed streptavidin-mediated pull-down of cross-linked, fragmented chromatin. These experiments were performed in cells expressing only BirA or in the two single cell clones expressing BirA together with the Flag/biotin-tagged TFII-I (clones 5 and 18). The isolated DNA was subjected to high-throughput sequencing using the Illumina platform. For cells expressing biotinylated TFII-I (Clone 5 and 18) we obtained 6.7 and 5.8 million reads, respectively, and for cells expressing only BirA we obtained 7 million reads. Given the very high correlation observed between sequencing results obtained by the two independent bioTFII-I ChIP sequencing experiments (Figure 2A), we merged them into one data set. Using the MACS algorithm (31), we assembled the unique, non-redundant sequence reads into peaks that identify potential TFII-I bound regions across the genome. This approach yielded a total of 18 920 potential TFII-I binding sites in K562 cells. Each TFII-I peak was assigned to its nearest gene, thus identifying a total of 5886 potential TFII-I target genes in K562 cells. Location analysis of identified peaks revealed selective binding of TFII-I in intergenic (∼68%) rather than intragenic (∼31%) regions and a very small fraction of peaks directly overlapping gene transcription start or end sites (∼1%, transcription start site [TSS] and transcription end site [TES], respectively) (Figure 2B). These findings are in accordance with the results of previous ChIP-chip experiments in mouse ES cells for TFII-I (5,22).

In order to assay for the expression state of the potential TFII-I target genes identified, we used the gene expression levels, determined by mRNA sequencing in K562 cells, available from the Encode project (GSM5916660) (33,34). Based on this analysis and using a ‘Reads per kilo base per million’ (RPKM) cut-off value of 1, a total of 2994 (∼51%) TFII-I bound target genes are expressed, whereas 2893 (∼49%) are silent in K562 cells (Figure 2C), thus showing no overt preference for TFII-I binding to either active or inactive genes. This lack of association between gene expression and TFII-I binding is reminiscent of previous ChIP-chip studies performed in mouse ES cells (5,22).

Even though there is no clear relationship between TFII-I binding and target gene expression, if we compare the occupancy profiles of TFII-I between expressed and silent target genes, some distinct patterns begin to emerge (Figure 2D). The distribution of peaks mapping proximally (±2 kb from TSS) to expressed genes (green line) differs from that of silent genes (red line). In expressed genes TFII-I preferentially binds upstream of the TSS, whereas at silent genes, TFII-I peaks are more evenly distributed upstream and downstream of the TSS. Overall, TFII-I binding downstream of the TSS of repressed genes is more abundant compared to expressed genes (*P* = 0.0026, chi-square test). Additionally, in both upstream and downstream binding events, TFII-I peaks associated with active genes are closer to the TSS compared to silent genes (Supplementary Figure S1, *p*_upstream_ = 7e-4, *p*_downstream_ = 6e-4, Wilcoxon rank sum test).

**Figure 3.**
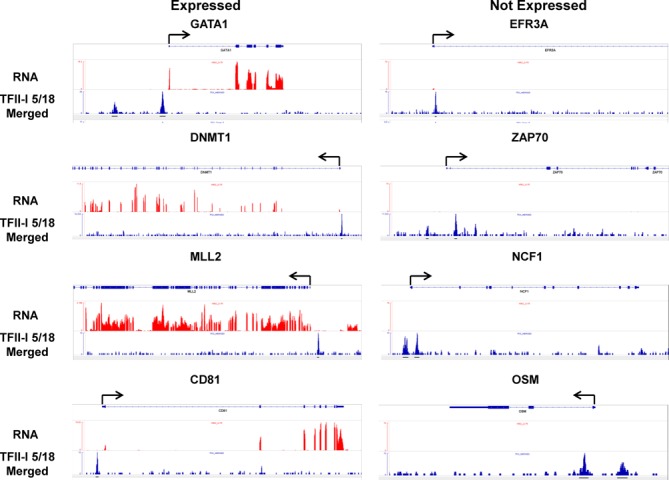
TFII-I occupancy at four expressed and four silent genes in K562 cells. Transcription (RNA) and TFII-I binding peaks at the expressed *GATA1*,*DNMT1,MLL2* and *CD81*, as well as the non-expressed *EFR3A*, *ZAP70*, *NCF1* and *OSM* genes. The scale refers to read counts that were normalized using the peak calling algorithm (MACS). Arrows indicate the direction of transcription. The results shown for TFII-I represent the combined data of clones 5 and 18.

The differential binding patterns of TFII-I at active or repressed genes is illustrated with a few examples in Figure 3. The genes encoding for GATA-1, DNMT1, MLL2 and CD81 are all expressed in K562 cells as revealed by the ENCODE RNA seq. (33,34). In each case, TFII-I binding was strongly associated with sequences immediately upstream of the TSS. On the other hand, genes encoding for EFR3A, ZAP70, NCF1 and OSM, which are expressed near or below detection in K562 cells, revealed peaks of TFII-I associations either only downstream (*EFR3A*) or upstream and downstream (*NCF1*, *OSM* and *ZAP70*) of the TSS.

**Figure 4.**
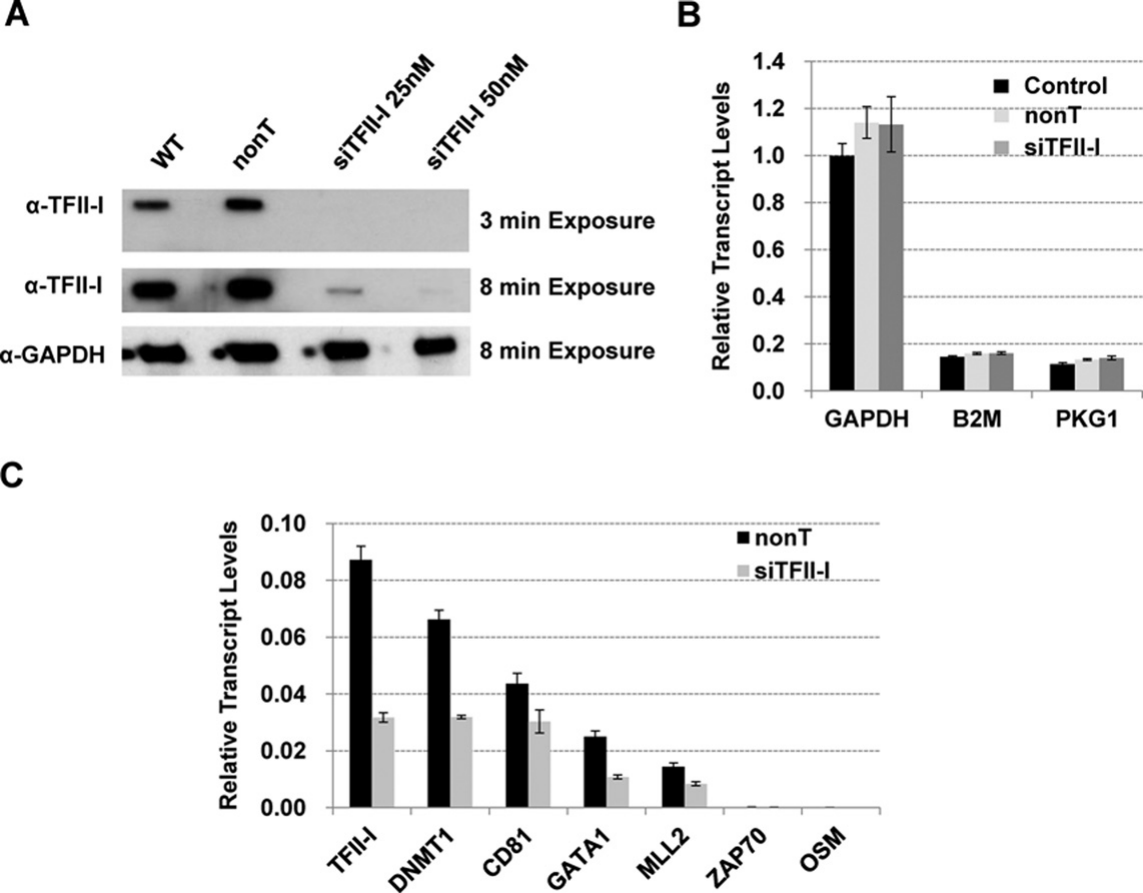
Functional analysis of TFII-I with respect to selected target genes in K562 cells. (A) Western blot analysis of TFII-I expression in wild-type (WT) K562 cells and in K562 cells transfected with siRNA pools against TFII-I at different concentrations (25 or 50 nM) or a non-targeting pool of siRNAs (nonT). Analysis of GAPDH served as a protein loading control. (B) Analysis of GAPDH, B2M and PgK1 gene expression in WT K562 cells or K562 cells transfected with siRNA pools against TFII-I (TFII-I at 50 nM) or non-targeting (nonT) siRNA pools. RNA was extracted, reverse transcribed and subjected to qPCR. (C) Analysis of TFII-I target gene expression in K562 cells transfected with siRNA pool against TFII-I (siTFII-I) or with non-targeting siRNA (nonT). RNA was extracted from the cells, reverse transcribed and subjected to qPCR using primers specific for the TFII-I, DNMT1, CD81, GATA1, MLL2, ZAP70 and OSM. Data in (B) and (C) were normalized to expression of GAPDH and the data represent two independent experiments with the qPCR performed in triplicate +/− SEM. Statistical analysis was based on the students *t*-test with *P* < 0.05 for all differences observed between nonT and siTFII-I data in (C).

To functionally characterize the role of TFII-I in gene expression, we inhibited expression of TFII-I by siRNA (Figure 4A). We focused on 6 genes for which we provided the binding patterns of TFII-I in Figure 3. Figure 4A demonstrates successful inhibition of TFII-I expression in cells transfected with the TFII-I siRNA pool compared to cells transfected with the control siRNAs (nonT). Expression of the *GAPDH*, *B2M* and *PgK1* genes, which did not associate with TFII-I, were not affected by reduced levels of TFII-I (Figure 4B). The mRNA levels of four expressed genes (*DNMT1*, *CD81*, *GATA1* and *MLL2*) decreased in cells expressing diminished levels of TFII-I compared to cells transfected with SC siRNAs (Figure 4C). Consistent with the ENCODE data (33,34), the *ZAP70* and *OSM* genes were expressed at or below detection and the data show that inhibition of TFII-I activity was not sufficient to increase expression of these genes. These data provide validation for the genomic TFII-I binding analysis presented above. Gene ontology (GO) analysis did not reveal any particular functional association of TFII-I target genes. More importantly, there was no meaningful functional distinction between expressed and silent TFII-I targets in the individual clusters of genes (data not shown). This is in contrast to GO ontology classification of TFII-I target genes in mouse ES cells which identified enrichment for chromatin assembly, cell fate commitment and signaling pathway ontologies (5). This contrast suggests differential TFII-I functions in mouse ES cells versus committed human hematopoietic cells.

**Figure 5.**
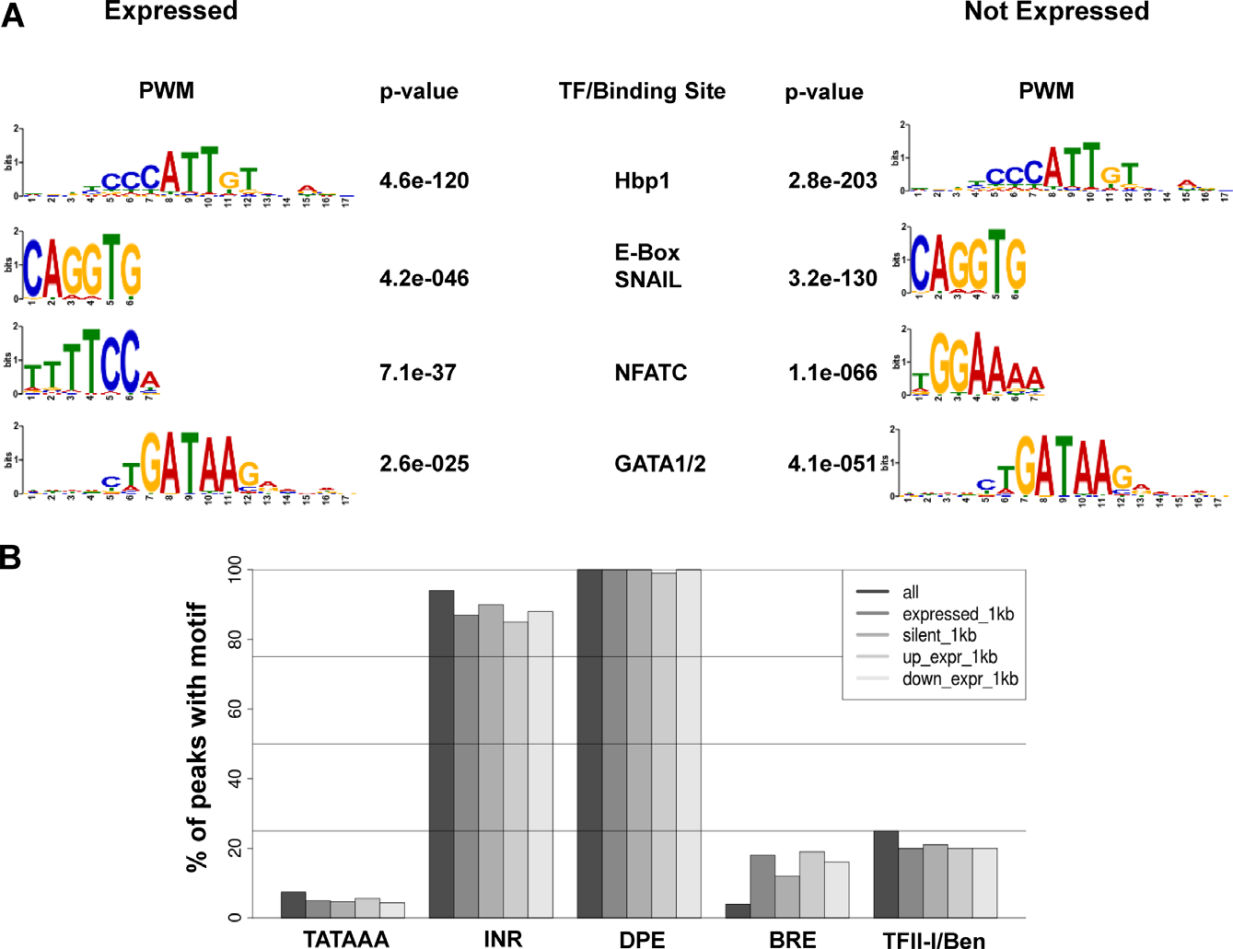
Analysis of TF binding sites associated with TFII-I peaks. (A) Consensus sequences associated with TFII-I binding peaks (PWM: position weight matrix) and corresponding TFs in expressed and silent (Not Expressed) genes. (B) Association of basal promoter elements and known TFII-I/Ben binding sites with TFII-I peaks within 1 kb of the TSS in expressed and silent genes. Also shown are whether the peaks within 1 kb are upstream or downstream of the TSS for the expressed subset of genes. The following sequence elements were examined; TATAAA (TATA-box), Initiator (INR, YYANWYY), Downstream Promoter Element (DPE, RGWYV), TFII-B recognition element (BRE, SSRCGCC) and the TFII-I/Ben binding site (RGATTR).

In order to identify putative DNA consensus sequences associated with TFII-I binding sites we used the MEME-ChIP algorithm (38), to perform *de novo* motif analysis on the sequences underlying the TFII-I peaks (Figure 5A). A region of 75 nucleotides around the summit of each TFII-I peak that map within 10 kb or less from an annotated gene TSS, was used as input in all subsequent analyses. Peaks assigned to either expressed or silent TFII-I targets returned nearly identical results (Figure 5A) with the most enriched consensus being related to the HMG-box protein 1 (Hbp1) consensus sites. E-box and GATA motifs as well as binding sites for NFAT TFs were also found to be highly enriched in both data sets. Discriminative motif analysis between peaks assigned to expressed or silent genes did not produce any notable differences.

In addition to the unbiased approach of the *de novo* analyses, we performed a more targeted approach searching for all consensus sequences associated with core RNA Pol II promoters including the TATA box, Initiator (INR), Downstream Promoter Element (DPE), TFII-B recognition element (BRE) and the TFII-I and BEN SELEX identified consensus (RGATTR) (2,5) (Figure 5B). Peaks over 1 kb upstream or downstream of a gene's TSS were excluded from this analysis. Again, peaks assigned to expressed or non-expressed genes produced similar results, except for differences in the BRE element. Interestingly, the BRE element is overrepresented in TSS-proximal TFII-I peaks compared to all TFII-I peaks (*P* < 2.2e-16, chi-square test). This is in contrast to all other core RNA Pol II promoter elements which show no preferential association with TSS-proximal peaks (Figure 5B). Furthermore, there appears to be a preferential association of TSS proximal TFII-I associated BRE motifs with active genes (*P* = 0.0006, chi-square test). These data suggest that TFII-I can distinguish BRE motifs depending on their proximity to TSS, and to a lesser extent, on their transcriptional status. Moreover, GO analysis of BRE-containing active genes with a TFII-I peak within 1 kb upstream of the TSS (‘up expr 1 kb’ in Figure 5B) shows enrichment for genes implicated in stress response (Table 1).

**Table 1.** Enriched Gene Ontologies in genes with TSS upstream and proximal TFII-I peaks as well as harboring a BRE element (80 genes)

Category	Term	Count	%	*P*-value	Genes
GO:0033554	cellular response to stress	8	10	0.018	BAZ1B, FOXA3, DUSP10, DDB2, GTF2H4, PKN1, CEP164, SCAP
GO:0006412	translation	6	7.5	0.021	RSL1D1, TUFM, RPS14, EIF3F, EEF2K, WARS2
GO:0006368	RNA elongation from RNA polymerase II promoter	3	3.75	0.022	GTF2A2, GTF2H4, POLR2D
GO:0006354	RNA elongation	3	3.75	0.025	GTF2A2, GTF2H4, POLR2D
GO:0016050	vesicle organization	3	3.75	0.026	EPS15, ARF1, ZFYVE16
GO:0006367	transcription initiation from RNA polymerase II promoter	3	3.75	0.042	GTF2A2, GTF2H4, POLR2D

**Figure 6.**
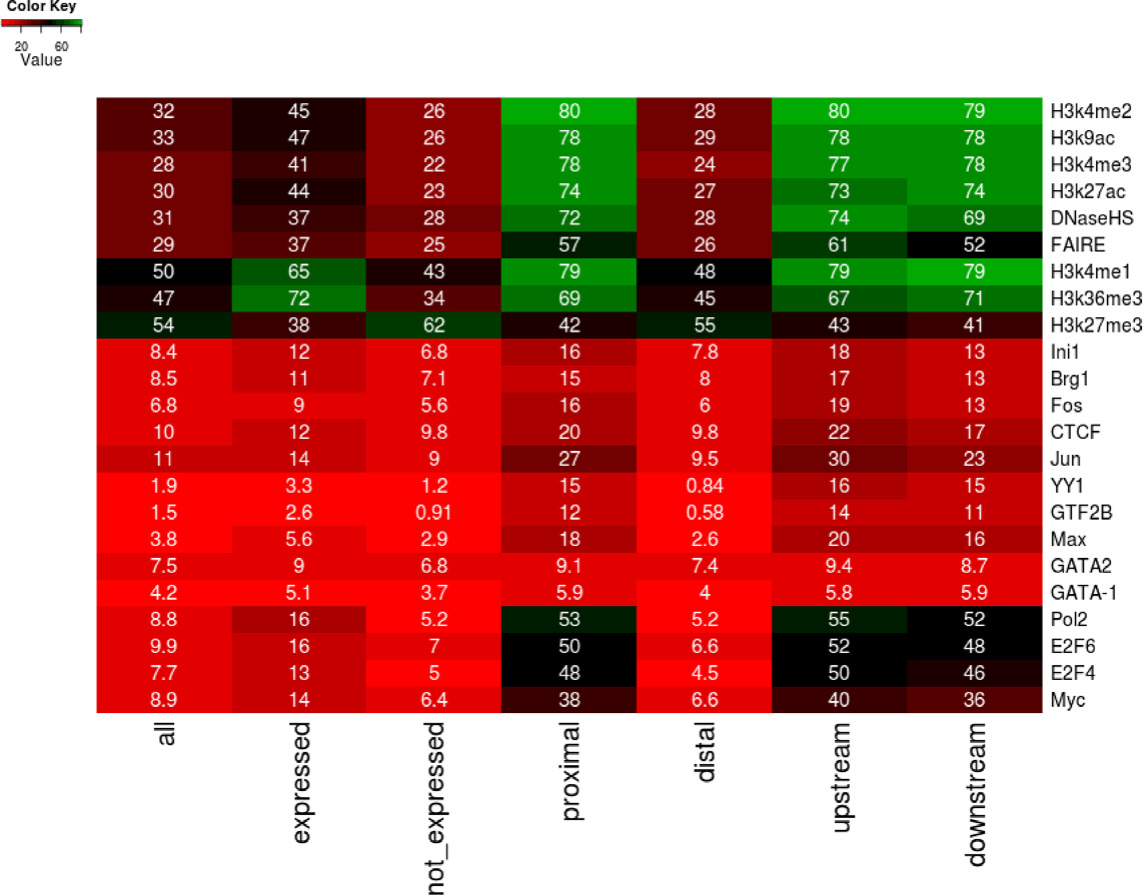
Association of TFII-I binding peaks with epigenetic marks or TF occupancy in K562 cells. Shown is a heatmap correlating TFII-I binding at various positions relative to the TSS of expressed and silent genes with epigenetic marks and TF binding peaks in K562 cells (high correlations are in green, lower correlations are in red). Numbers represent the percentage of TFII-I peaks that overlap with a specific histone tail modification or a TF-peak within a 150 nt window.

To further characterize the genomic loci occupied by TFII-I in K562 cells we computed their overlaps with publicly available genome-wide occupancy profiles provided by the Encode project (33,34) (Figure 6). TFII-I peaks were divided into different categories based on the expression state of their target genes (expressed [6309 peaks] or silent [12611 peaks]), their distance to the nearest TSS (proximal [±1 kb from TSS, 1413 peaks]) or distal (over 1 kb from TSS [17438 peaks]), and proximal peaks were further divided with respect to the target gene's TSS as upstream (770 peaks) or downstream (643 peaks). Results are presented as a heatmap in Figure 6 and represent the percentage of TFII-I peaks that overlap with a specific histone tail modification or TF peak within a 150 nt window. Consistent with current models of gene regulation, TFII-I peaks associated with expressed genes show a significant overlap with activating histone marks (H3K4me2, H3K4me3, H3K9Ac, H3K27Ac, H3K4me1 and H3K36me3), whereas TFII-I peaks associated with silent genes present the highest overlap with the H3K27me3 silencing histone mark. Additionally, we found a very high preference of proximally bound TFII-I peaks for specific TFs with respect to distal TFII-I peaks. More specifically, we found significantly high overlap of TFII-I with the E2F4 and E2F6 factors (∼50%) in proximal peaks, followed by a series of E-box binding TFs, such as c-Myc, Max, as well as c-Jun and c-Fos. TF c-Myc interacts with E-box sequences (CANNTG), which are also bound by USF proteins. This supports previous findings showing that TFII-I interacts with c-Myc and USF at initiator or E-box elements (15,16). The co-binding of E2Fs and TFII-I is interesting in light of recent data showing that E2Fs associate with the majority of active promoters in B-lymphocytes (39). Overlap with GATA factors did not seem to specifically associate with any subset of TFII-I peaks. Distal TFII-I peaks seemed to be marked by the silencing H3K27me3 mark or the enhancer-associated H3K4me1 histone mark.

### TFII-I interacts with topoisomerase II α and β, SWI/SNF chromatin remodeling complexes, TAF15 and Elongin A

**Figure 7.**
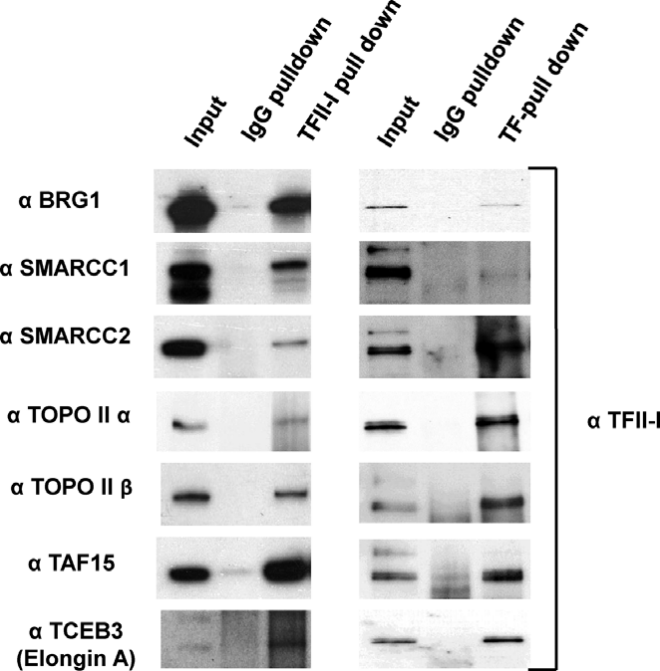
Analysis of TFII-I interacting proteins by Co-IP. Nuclear extracts from K562 cells were subjected to immunoprecipitation using IgG control or TFII-I antibodies. Proteins from the input (before IP), IgG pull-down and TFII-I pull-down were fractionated by SDS-PAGE and subjected to western blot analysis using the antibodies shown on the left or antibodies specific for TFII-I (right panel).

We performed a preliminary Mass-spec analysis of proteins associated with TFII-I in clones 5 and 18. The cell clones 5 and 18 as well as the control cells expressing only the BirA ligase were expanded and subjected to large scale nuclear protein extraction. The nuclear extracts were then incubated with streptavidin-coated magnetic beads. After several rounds of washing, as described in Materials and Methods, proteins were eluted and either fractionated by SDS-PAGE (Figure 1E) or directly subjected to trypsinization ‘on-the-beads’. Protein samples were then analyzed by Orbi-Trap Mass-Spec. In several experiments we identified TFII-I, components of SWI/SNF chromatin remodeling complexes, including Brg1, SMARCC1 and SMARCC2, topoisomerases II α and β, the TBP-associated factor (TAF) 15 and transcription elongation factor Elongin A. We confirmed interactions between the proteins and TFII-I using stringent Co-IP experiments with nuclease treated nuclear extracts (Figure 7). The interactions with Brg1, SMARCC1, SMARCC2, Topo IIα, Topo IIβ, TAF15 and Elongin A (TCEB3) were confirmed by both TFII-I immunoprecipitation as well as reverse immunoprecipitations with antibodies against TFII-I protein interacting partners (Figure 7). Some of the proteins showed weak bands in the western blotting experiments which could be due to the fact that only a small fraction of TFII-I associated with these proteins or that the interactions are unstable. Other proteins, like TAF15, showed robust signals suggesting more abundant interactions (Figure 7).

**Figure 8.**
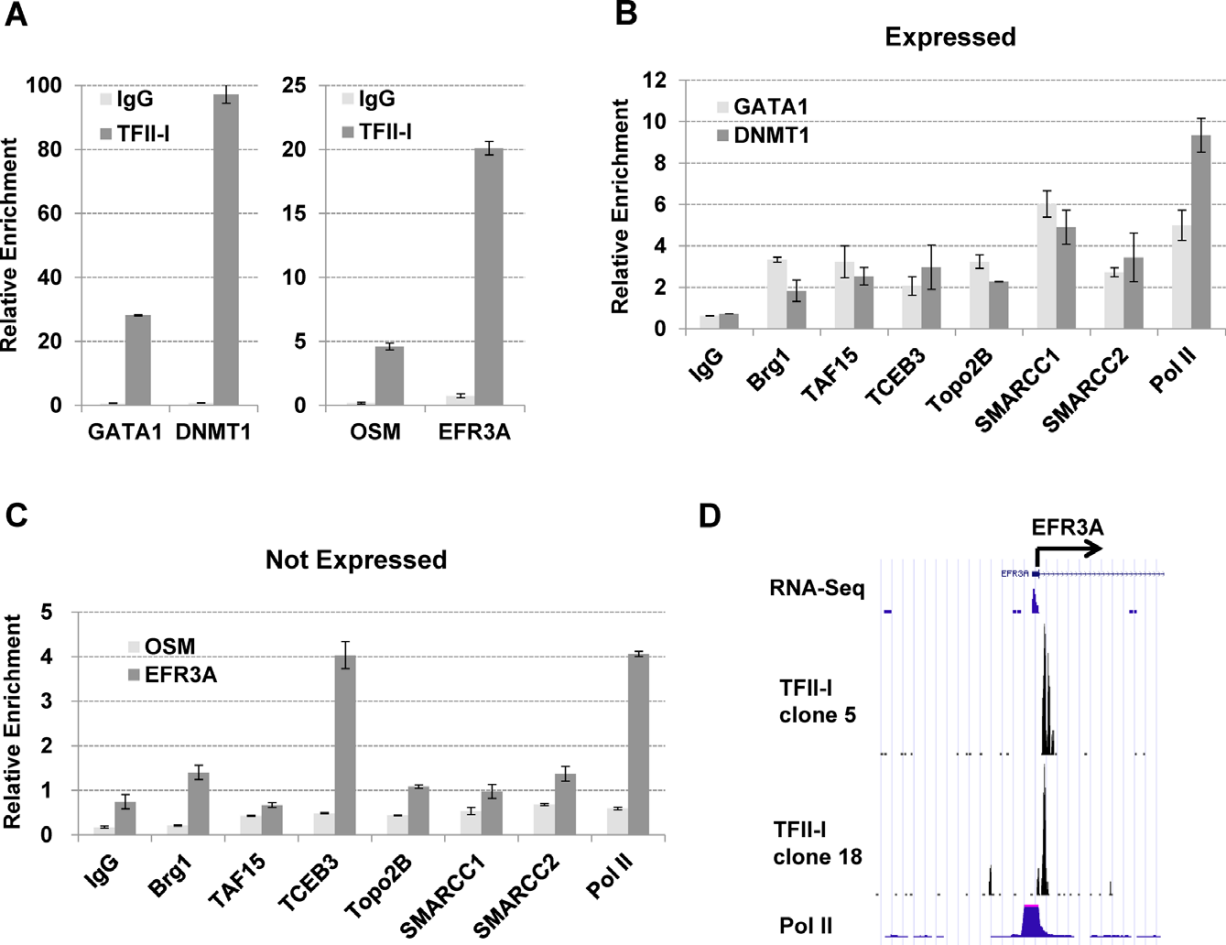
Analysis of the occupancy of TFII-I and associated proteins with two expressed and two silent genes in K562 cells. (A, B, C) K562 cells were subjected to ChIP assays using the antibodies shown at the bottom of the graphs. The precipitated DNA was subjected to qPCR using primers specific for the expressed *GATA1* and *DNMT1* (A and B) and the silent *OSM* and *EFR3A* (A and C) genes as indicated. The data were normalized to the HS2–3 region and represent the results of two independent experiments with the qPCR performed in triplicate +/− SEM. All data in (B) with respect to binding of TFs to the *GATA1* and *DNMT1* genes compared to IgG were statistical significant (*P* < 0.05) according to student's *t*-test. With respect to (C) binding of all the factors to the *OSM* gene, except Brg1, compared to IgG revealed statistical significance according to the student's *t*-test (*P* < 0.05). The same is true for binding to the *EFR3A*, except for TAF15 and SMARCC1. (D) TFII-I and Pol II occupancy at the *EFR3A* promoter in K562 cells. The scale refers to read counts normalized using the peak calling algorithm (MACS). The arrow indicates the direction of transcription.

Next, we performed ChIP experiments to (i) verify that TFII-I associates with selected target genes outlined in Figure 4 (see Supplementary Figure S2A) and (ii) to examine if TFII-I associated proteins co-occupy the same chromosomal regions together with TFII-I (Figure 8). Supplementary Figure S2A shows that TFII-I associated with 8 gene loci at which we detected peaks in the high-throughput sequencing analysis, including GATA1, DNMT1, CD81–5, MLL, EFR3A, ZAP70, OSM and ATF3. We next focused our attention to four gene loci, two that are expressed (DNMT1 and GATA-1), and two that are expressed at low levels in K562 cells (OSM and EFR3A). As a negative control we examined a DNAse I insensitive region in the human β-globin gene locus that is located in between two β-globin gene locus control region hypersensitive sites (HS2–3) and not known to interact with TFs (33,34). All ChIP data were normalized to the β-globin locus control region HS2–3 segment. The data demonstrate that endogenous TFII-I associated with all of the gene loci identified by the streptavidin-pull-down/high-throughput sequencing experiment (Figure 8A and Supplementary Figure S2). Furthermore, all of the proteins that were shown to interact with TFII-I in the Co-IP experiments associated together with TFII-I at the expressed *GATA1* and *DNMT1* gene loci (Figure 8B). At the silent *OSM* gene, we only detected moderate binding of TFII-I (Figure 8A). Interestingly, however, the *EFR3A* gene associated with TFII-I, Pol II, and Elongin A (TCEB3; Figure 8A and C). Comparison of the TFII-I binding peak with the binding of Pol II and RNA-seq-data, retrieved from the K562 ENCODE project data, revealed that TFII-I associated immediately downstream of a Pol II peak at the *EFR3A* promoter (Figure 8D). To analyze the co-localization of TFII-I with TAF15, Elongin A and Pol II in more detail we examined three different regions in the DNMT1 and EFR3a gene loci by ChIP (Supplementary Figure S2B). The data demonstrate that TAF15, Elongin A (TCEB3), and Pol II localized together with TFII-I at the TFII-I peak in the DNMT1 promoter. None of these proteins revealed significant binding at regions 1 kb upstream or downstream of the TFII-I peak compared to IgG pull down. At the EFR3A region TFII-I, Elongin A and Pol II associated with the TFII-I peak but not with a region 1 kb upstream. We did not detect TFII-I binding at a region 1 kb downstream of the TFII-I peak and the binding of Elongin A and Pol II were significantly reduced at this site. Binding of TAF15 was not detected in the EFR3A gene locus (Supplementary Figure S2), presumably due to the low transcriptional status in K562 cells.

**Figure 9.**
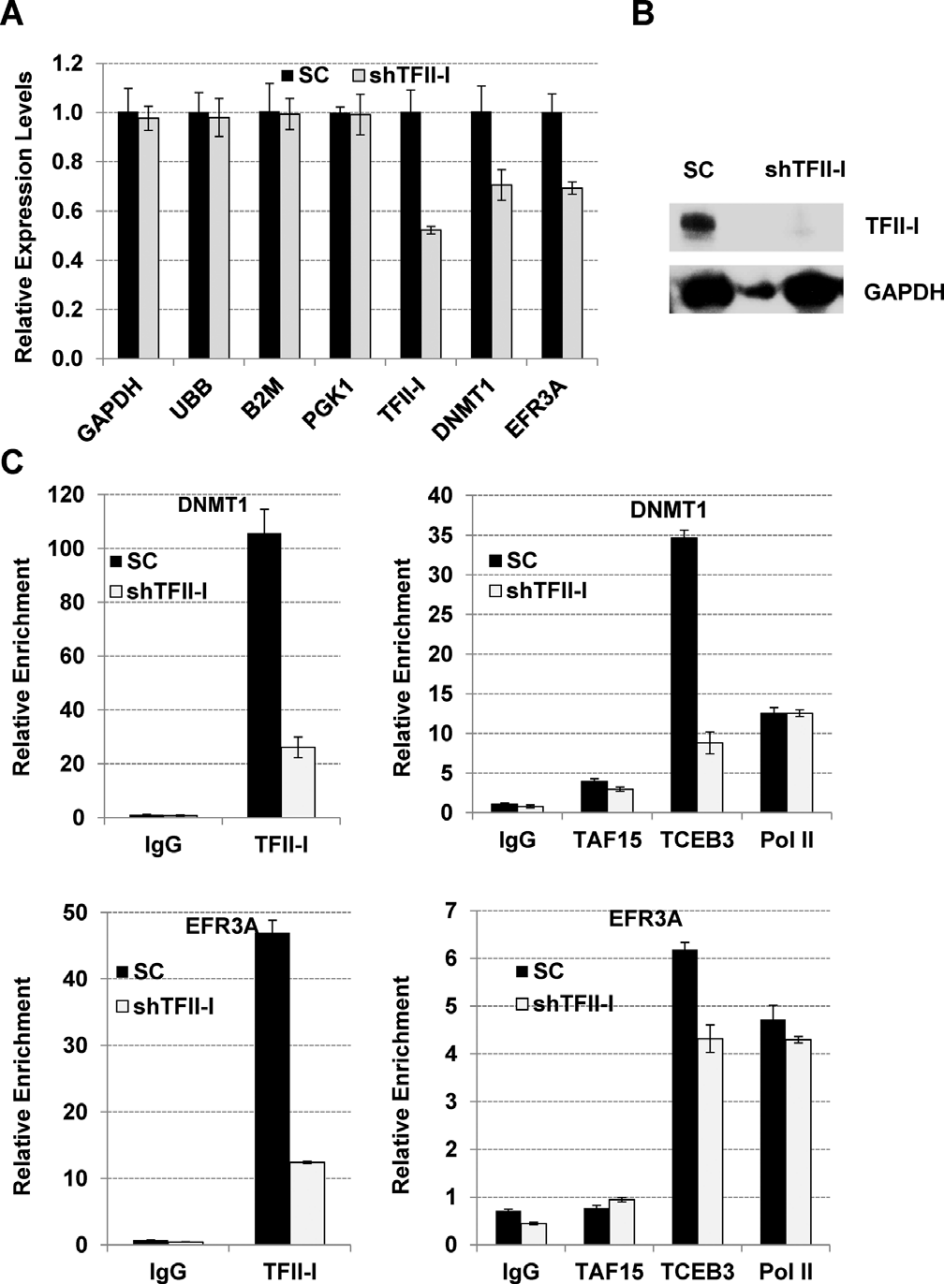
Reduced association of Elongin A at the DNMT1 and EFR3A gene loci in K562 cells expressing TFII-I directed shRNA. (A) K562 cells expressing TFII-I shRNA (shTFII-I) or SC were subjected to expression analysis using primers specific for the GAPDH, UBB, B2M, PGK1, TFII-I, DNMT1 and EFR3A genes. Relative expression levels, with levels in SC cells set at 1, are shown. The data represent the results of two independent experiments with the qPCR performed in triplicate +/− SEM. Reduced expression of TFII-I, DNMT1 and EFR3A was statistically significant (*P* < 0.05). (B) Western blot analysis of TFII-I and GAPDH expression in K562 cells expressing TFII-I shRNA (shTFII-I) or SC. (C) TFII-I, Elongin A (TCEB3), and Pol II occupancy at the DNMT1 and EFR3A gene loci in K562 cells expressing TFII-I shRNA (shTFII-I) or SC. K562 cells were subjected to ChIP and the purified DNA was analyzed by qPCR using primers specific for the TFII-I binding peak at the DNMT1 and EFR3A gene loci. The data represent the results of two independent experiments with the qPCR performed in triplicate. Data showing reduced association of TFII-I and Elongin A (TCEB3) with the DNMT1 and EFR3A genes were statistically significant (*P* < 0.05).

We next analyzed if reduced expression of TFII-I affects the association of TAF15, Elongin A and Pol II at the DNMT1 and EFR3A gene loci (Figure 9). We generated single K562 cell clones expressing shRNA directed against TFII-I (shTFII-I) or SC shRNA. The data show that TFII-I expression at the RNA level was reduced by about 50% in the shTFII-I clone compared to the control, whereas the reduction of TFII-I protein level appeared to be greater (Figure 9A and B). The decrease in TFII-I expression was associated with reduced expression of DNMT1 and EFR3A, but had no effect on expression of the housekeeping genes GAPDH, UBB, B2M and PGK1. At both the DNMT1 and the EFR3A genes, reduced expression of TFII-I led to a decrease in the association of Elongin A (TCEB3, Figure 9C). However, the binding of TAF15 and Pol II was unperturbed at these genes in cells expressing shTFII-I compared to control cells (SC, Figure 9C). These data suggest that TFII-I regulates at least some genes at the level of transcription elongation.

### TFII-I associates with several genes involved in response to cellular stress and binds immediately downstream of Pol II peaks

**Figure 10.**
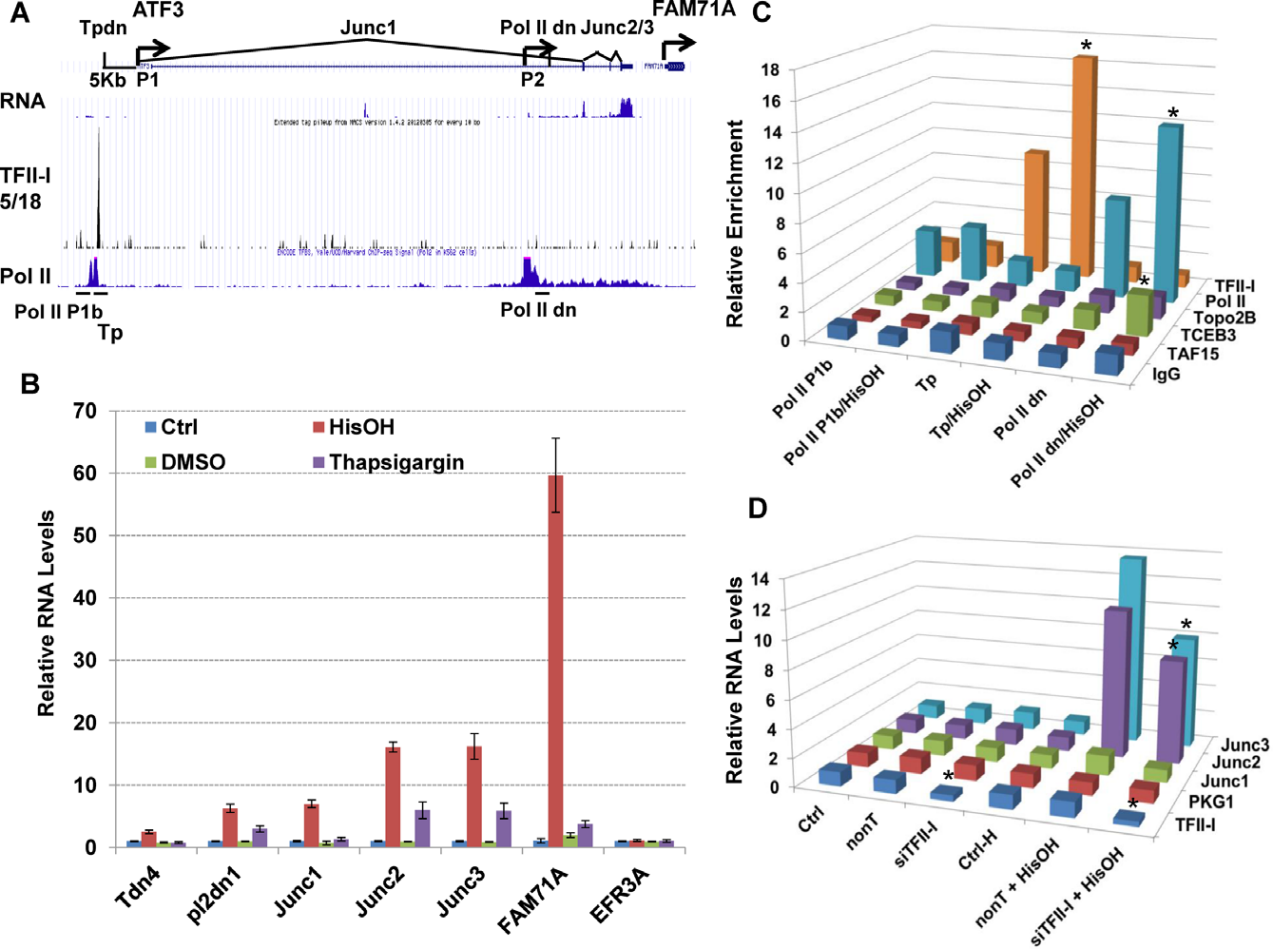
TFII-I binds immediately downstream of a Pol II peak in the *ATF3* gene locus and nutrient stress increased transcription downstream of the TFII-I/Pol II peak and across the ATF3 gene locus. (A) TFII-I and Pol II occupancy at the *ATF3* and *FAM71A* gene loci in K562 cells. The scale refers to read counts normalized using the peak calling algorithm (MACS). The arrows indicate the direction of transcription at the two *ATF3* promoters (P1 and P2) and the *FAM71A* promoter. Regions analyzed by RT-PCR to assess RNA abundance are indicated on top of the locus, and regions analyzed by ChIP are indicated at the bottom. (B) Analysis of *ATF3*, *FAM71A* and *EFR3A* gene expression in K562 cells and in K562 cells treated for 8 h with 2 mM histidinol (HisOH) or 100 nM thapsigargin as indicated. Several regions of the *ATF3* gene locus were analyzed including a region downstream of the TFII-I binding peak (Tpdn), exon/exon junctions 1, 2 and 3 (Junc1, 2 and 3), and a region downstream of promoter P2 (Pol II dn). RNA was extracted and analyzed as outlined in the legend to Figure 5. The data were from two independent experiments with the qPCR performed in triplicate. Changes in gene expression across the ATF3 and FAM71A gene locus in response to HiOH treatment revealed statistical significance according to student's *t*-test (*P* < 0.05). (C) ChIP analysis of TFII-I, TAF15, TCEBP3 (Elongin A), Topo II β and Pol II, at the *ATF3* gene locus in K562 cells or K562 cells treated for 8 h with 2 mM HisOH. Cross-linked chromatin was sonicated and precipitated with the respective antibodies, including as a negative control IgG. DNA was isolated from the precipitate and subjected to qPCR using primers specific for the *ATF3* gene locus; Tp, corresponding to the TFII-I peak; Pol II P1b, a region corresponding to the upstream Pol-II/TFII-I binding peak and Pol II dn, corresponding to a transcribed region of the *ATF3* gene. Data were normalized to a region in the human β-globin LCR (HS2/3) and represent the results of two independent experiments with the qPCR performed in triplicate. Increased binding of Pol II to Pol IIP1b and Pol IIdn, as well as increased binding of TFII-I to Tp, and TCEB3 (Elongin A) to Pol II dn revealed statistical significance (*P* < 0.05). (D) ATF3 (Junc 1, 2 and 3, as described in panel B) and control mRNA (TFII-I, PKG1) analysis in K562 cells treated with 25 nM TFII-I siRNA (siTFII-I) or non-targeting siRNA (nonT) in the presence or absence of histidinol (HisOH, as described in panel B). The data were from two independent experiments with the qPCR performed in triplicate. Changes in gene expression of ATF3 (Junc2 and Junc3) and TFII-I in TFII-I siRNA and/or HisOH treated cells, compared to nonT-siRNA treated cells revealed statistical significance according to student's *t*-test (*P* < 0.05). Data with error bars for (C) and (D) are presented in Supplementary Figure S5.

Given that some of the proteins found to associate with TFII-I (TAF15 and Elongin A) are known to be involved in gene regulation during cellular stress (40,41), we examined the binding of TFII-I to several stress responsive genes. TFII-I interacted with genes encoding for the heme-regulated eIF2α kinase HRI (*EIF2AK1*), the Cockayne syndrome-B (*CS-B* or *ERCC6*), heterochromatin protein 1 beta (Hp1 beta, *CBX1*) and TF ATF3 (Supplementary Figure S3). TFII-I bound at these stress responsive genes at their 5′ ends and the genes revealed a peak of Pol II binding upstream of the TFII-I peak. We focused our attention to TF ATF3, which is induced in response to amino acid limitation and endoplasmic reticulum stress (42,43). Previous studies have shown that ATF3 expression is controlled by two alternative promoters, P1, and P2 (Figure 10A) (41–44). Promoter P1 is utilized in some cancer cell lines (44), while P2 appears to be the dominant promoter in most cell-types (42,43). Based on the ENCODE data, ATF3 is transcribed at very low levels in K562 cells mediated by promoter P2 (Figure 10A). The interaction of TFII-I with a region 5 kb upstream of promoter P1 of the *ATF3* gene was striking and did not appear to fit the previous observations that TFII-I interacted with regions downstream of the TSS in repressed genes (Figure 10A). Interestingly, similar to what we observed at the repressed *EFR3A* gene, a relatively large peak representing Pol II binding is immediately upstream of the TFII-I binding peak within the ATF3 gene locus. The presence of H3K27ac, H3K4me, Pol II and other marks indicate that the TFII-I bound region is an enhancer element (Supplementary Figure S4). We next cultured K562 cells in the presence of histidinol (HisOH) to mimic amino acid limitation or with thapsigargin to induce endoplasmic reticulum stress and then examined several regions across the ATF3 gene locus for RNA production. As shown in Figure 10B, expression of the ATF3 mRNA, as determined by using primers spanning the junctions of exons 2/3 and exons 3/4, (Junc2 and Junc3), increased by more than 16-fold in cells treated with 2 mM HisOH for 8 h compared to untreated cells. Importantly, we also detected increased transcription at a non-coding region downstream of the TFII-I/Pol II binding peak (Tpdn), but upstream of promoter P2, suggesting release of the paused Pol II. Treatment of K562 cells with 100 nM thapsigargin for 8 h led to a 5-fold increase of ATF3 transcription (Junc2 and Junc3). Expression of the *EFR3A* gene remained unaltered in the presence or absence of HisOH or thapsigargin demonstrating that stimulation of elongation of Pol II at this promoter required different stimuli (Figure 10B). Expression of the *FAM71A* gene which is located in close proximity downstream of the *ATF3* gene was upregulated by almost 60-fold in response to HisOH treatment. Treatment with thapsigargin did not significantly enhance FAM71A expression over the Dimethyl Sulfoxide (DMSO) treated control cells.

Next, we examined the binding of TFII-I, Pol II, Topo IIβ, Elongin A and TAF15, with three specific regions in the ATF3 gene locus before and after induction of expression with HisOH (Figure 10C). We restricted the analysis to HisOH treated cells as we detected more robust induction of ATF3 under these conditions. At the Pol II peak upstream of TFII-I binding (Pol II P1b), we detected only Pol II and low levels of TFII-I. At the TFII-I peak (Tp) we detected only TFII-I, and HisOH treatment increased the binding of TFII-I but did not change association of other proteins in this region. We also examined a region downstream of a Pol II peak associated with promoter P2 (Pol II dn) and found that the association of Pol II increased with this region after incubating K562 cells with HisOH (Figure 10C). Importantly, Elongin A was only detectable at the ATF3 gene locus in HisOH treated cells and the association of Elongin A was restricted to the promoter P2 controlled coding region of ATF3. These data suggest that Elongin A promotes enhanced expression of ATF3 from promoter P2 in the presence of cellular stress, consistent with published data (41,42). Because we showed that TFII-I interacts with Elongin A (Figure 7) it is possible that there is an interaction between the TFII-I bound enhancer and promoter P2 that delivers Elongin A to the ATF3 coding region. This hypothesis is supported by ChIA-Pet data, available from the ENCODE Project Consortium (33,34), that reveal interactions between the TFII-I/Pol II peak upstream of promoter P1 with the promoter P2 in K562 cells (Supplementary Figure S5).

Inhibition of TFII-I expression by siRNA did not change the basal expression levels of the ATF3 gene in K562 cells (Figure 10D and Supplementary Figure S6). However, the reduction in TFII-I expression decreased the induction of ATF3 gene transcription by HisOH. These results show that TFII-I is required for the stress-induced expression of the ATF3 gene, which could be mediated by delivery of Elongin A to the P2 promoter via a looping mechanism.

## DISCUSSION

In many ways TFII-I is an unusual TF. It contains multiple R-repeats that resemble helix-loop-helix proteins, it functions in both cytoplasm and nucleus, and it functions as an activator and repressor of transcription (1,2). TFII-I appears to regulate a large number of genes including housekeeping and tissue-specific genes as well as genes that code for cell-cycle regulators or proteins involved in response to cellular stress. In this study, we identified TFII-I interacting proteins and target DNA sequences in human erythroleukemia cells. We chose to perform these studies in K562 cells as these cells have been extensively analyzed in the context of the human ENCODE project (33,34). The ENCODE project provides genome wide data of chromatin associations of a variety of proteins and histone marks involved in the regulation of chromatin structure and transcription.

The genome-wide association analysis revealed that TFII-I interacted with intragenic as well as intergenic regions. Similar to what was reported by Bayarsaihan *et al.* for TFII-I interactions in ES cells and embryonic tissues (5,22), we did not find a clear correlation between TFII-I binding and the expression levels of genes located close to TFII-I binding sites in K562 cells. Comparison with the ENCODE data revealed that TFII-I binding peaks at silent genes was associated with repressed histone marks like H3K27me3, whereas peaks close to expressed genes were associated with histone marks typically found in active chromatin like H3K4me3 at proximal sites or H3K4me1 at distal sites. We identified three components of SWI/SNF chromatin remodeling complexes as TFII-I interacting proteins, including the ATPase Brg1 and associated proteins SMARCC1 and SMARCC2. Brg1 containing chromatin remodeling complexes have been associated with both activation and repression of transcription (45), similar to what has been reported for TFII-I. However, in this study we only found Brg1 associations with TFII-I bound to active genes. Previous studies found that Topo II α and β interact with Brg1 containing SWI/SNF chromatin remodeling complexes (45). The data suggest that TFII-I recruits large chromatin remodeling complexes including Topo II activity to promoters and other DNA regulatory elements.

One interesting aspect of the current study is the observation that TFII-I preferentially binds upstream of the TSS in expressed genes, and downstream, or upstream and downstream, of the TSS in repressed genes. A previous study by the Roeder laboratory found that TFII-I interacts with a downstream element in the IgH gene and that it functions as both repressor and activator at this site (23). Activation is mediated by the replacement of HDAC with Oca-B, which then mediates the interaction between the promoter and an enhancer. This or similar scenarios could also be envisioned for other repressed genes that have TFII-I bound downstream of the TSS (i.e. OSM, EFR3A, ZAP 70 and NCF1, Figure 3).

The analysis of DNA sequences common to the TFII-I peaks revealed some known and novel binding sites. For example, binding of TFII-I to E-box sequences has been shown before (16). It may be important that the current analysis identified E-box variants predicted to interact with the snail repressor proteins (46). Previous studies identified initiator (INR) and the downstream promoter element (DPE) as sites of TFII-I recruitment (1,2). We observed an enrichment for HMG-box protein (Hbp1) binding sites as well as binding sites for the TFs NFATC and GATA at TFII-I peaks. NFATC activity is regulated by calcium and cytoplasmic TFII-I inhibits the influx of calcium through interaction with PLC-γ (13,47). Thus, nuclear translocation of TFII-I may increase calcium influx and activation of NFAT TFs.

Bayarsaihan *et al.* identified additional TF sites associated with TFII-I binding events in ChIP/chip experiments in ES cells and embryonic tissues (5,22). It is possible that the focus on the TFII-I delta isoform in this study led to a more restricted number of binding sites (5,22). Analysis of TFII-I peaks with other DNA binding events in K562 cells revealed frequent association with the binding of E2F6 and E2F4.

Interestingly, while overall the GO analysis did not reveal specific clusters of genes regulated by TFII-I in K562 cells, we found that TFII-I peaks associated with stress responsive genes that correlated with increased presence of the BRE element (Table 1). Many stress responsive genes harbor paused RNA polymerase II at the promoter (48), and previous data have shown that TFII-I regulates stress responsive genes (1,2). It is thus possible that TFII-I regulates Pol II pausing and/or release from pausing at these genes. Indeed, we identified several genes, including the *ATF3*, *CS-B*, *CBX1* (Hp1 beta) and *EIF2AK1* genes, at which TFII-I binds immediately downstream of a Pol II peak (Supplementary Figure S3). Moreover, two components implicated in the response to cellular stress, Elongin A and TAF15 (40,41), were found to associate with TFII-I in this study.

TAF15, formerly known as TAF68, is, as the name implies, a TAF involved in basal Pol II transcription (49). TAF15 contains RNA binding domains and has been shown to interact with components of the spliceosome including the U1snRNP (50). Like Elongin A, TAF15 has been implicated in the cellular response to stress (40). In Co-IP experiments the interaction between TFII-I and TAF15 was found to be very strong. The interaction with TAF15 and the unusual binding patterns of TFII-I in expressed, repressed and inducible genes could hint to a new activity of TFII-I. Recently, the Sharp laboratory presented evidence that the directionality of transcription at many promoters is regulated by U1snRNP, which appears to protect sense transcripts from being cleaved by the polyadenylation and cleavage complex (51). Perhaps TFII-I and TAF 15 together recruit U1snRNP to the 5′ end of genes or to Pol II bound enhancer elements.

Elongin A is an activator of transcription elongation and has been shown to help Pol II overcome the paused state (52). In this respect, it is interesting to note that we found both TFII-I and Elongin A together with paused Pol II at the promoter of the repressed *EFR3A* gene. Elongin A interacts with two other components, Elongin B and C and, in addition to stimulating transcription elongation, Elongin A has been shown to recruit Elongin B/C and ubiquitinylating activities that lead to the degradation and removal of paused Pol II (53). Therefore, TFII-I and Elongin A could exert negative effects on transcription but could also be involved in activating transcription upon receiving specific stress signals. Interestingly, the association of Elongin A but not Pol II with the EFR3A gene requires TFII-I (Figure 9). Expression of the EFR3A gene is very low in K562 cells (33,34) and is further reduced upon reduction of TFII-I expression. Our data show that TFII-I is required for the recruitment of Elongin A to the EFR3A promoter and suggest that it does so by directly interacting with this elongation factor (Figure 7).

The bzip TF ATF3 has been implicated as a key regulatory molecule in pathological states as cancer (54) and the inflammatory response (55). ATF3 often serves as a transcriptional repressor, and has been shown to recruit multiple HDAC activities during autorepression of its own gene (56). The *ATF3* gene has been reported to have two promoters (P1 and P2, Figure 10A) separated by about 40 kb, which are regulated in a cell-type specific manner (44). The downstream promoter P2 has been more extensively studied and contains proximal regulatory elements that respond to a wide variety of extracellular stimuli, including hormones (57), non-steroidal anti-inflammatory drugs (58), amino acid limitation (59) and ER stress (43). TFII-I interacts with an element located 5 kb upstream of promoter P1 in the ATF3 gene locus and there is a binding peak for Pol II immediately upstream of the TFII-I site. This element exhibits characteristics of an enhancer including increased levels of H3K4me, H3K27ac and p300 (Supplementary Figure S4). Transcripts are detectable only upstream of the TFII-I binding peak suggesting that TFII-I prevents transcription elongation at this site (Figure 10A). Indeed, induction of transcription by HisOH increased transcription downstream of the TFII-I/Pol II peak and throughout the ATF3 gene locus. Induction of transcription did not cause the dissociation of TFII-I from the ATF3 gene locus, but rather increased its association suggesting that it is involved in both negative and positive aspects of *ATF3* gene transcription. This function would be similar to what has been described for TFII-I's role at an immunoglobulin gene locus (23). Importantly, HisOH induced higher levels of promoter P2-mediated transcription of the ATF3 coding region. The HisOH-mediated increase in transcription required TFII-I and correlated with enhanced association of Elongin A (TCEB3) with the ATF3 coding region. The Pol II ChIA-Pet data from The ENCODE Project Consortium (33,34) show that the TFII-I/Pol II bound region interacts with promoter P2 in K562 cells and, moreover, that it does not reveal any other interactions (Supplementary Figure S5). This strongly suggests that this element functions as an enhancer for promoter P2 in the ATF3 gene locus. Future studies will be directed toward testing the hypothesis that the upstream TFII-I bound enhancer associates with P2 and that Elongin A is recruited to this locus in a TFII-I dependent manner.

Taken together, our data reveal novel aspects of TFII-I protein–protein interactions and function that will guide our future studies on the association with other transcription regulators and the physiological consequences of their actions on specific genes. One focus will be to examine the functional relationship among TFII-I, Elongin A and TAF15 in controlling gene expression in response to cellular stress.

## SUPPLEMENTARY DATA

Supplementary Data are available at NAR Online.

## ACCESSION NUMBERS

The bioTFII-I data have been submitted to the Gene Expression Omnibus (GEO). The accession number is GSE51065.

## Supplementary Material

SUPPORTING INFORMATION
