# Supplementary material for: Genomic and proteomic analysis of transcription factor TFII-I reveals insight into the response to cellular stress
Source: Nucleic Acids Res. 2014 May 28;42(12):7625–41. doi: 10.1093/nar/gku467 (PMC4081084; doi:10.1093/nar/gku467)
Supplement: SUPPORTING INFORMATION [file supp_42_12_7625__index.html]

Genomic and proteomic analysis of transcription factor TFII-I reveals insight into the response to cellular stress — SUPPORTING INFORMATION 

# Genomic and proteomic analysis of transcription factor TFII-I reveals insight into the response to cellular stress

## SUPPORTING INFORMATION

**Files in this Data Supplement:**

- Supplemental Table and Figures
