# Supplementary material for: Genomic and proteomic analysis of transcription factor TFII-I reveals insight into the response to cellular stress
Source: Nucleic Acids Res. 2014 May 28;42(12):7625–41. doi: 10.1093/nar/gku467 (PMC4081084; doi:10.1093/nar/gku467)
Supplement: SUPPORTING INFORMATION [file supp_gku467_nar-01111-m-2014-File012.doc]

**Supplementary Table and Figures**

**Table S1: Encode Accession Numbers of Transcription Factors, Histone Modifications, DNase I hypersensitive sites, and Faire sequences.**

Table S2. Primer sequences.

| **Primer name** | **Sequence** |
| --- | --- |
| **ChIPseq Oligos** | |
| **MP_Adapt1** | **[Phos]GATCGGAAGAGCACACGTC*T** |
| **MP_Adapt2** | **ACACTCTTTCCCTACACGACGCTCTTCCGATC*T** |
| **Primer2_Index4** | **CAAGCAGAAGACGGCATACGAGATTGGTCAGTGACTGGAGTTCA**  **GACGTGTGCTCTTCCGATC*T** |
| **Primer2_Index5** | **CAAGCAGAAGACGGCATACGAGATCACTGTGTGACTGGAGTTCAG**  **ACGTGTGCTCTTCCGATC*T** |
| **Primer2_Index6** | **CAAGCAGAAGACGGCATACGAGATATTGGCGTGACTGGAGTTCAG**  **ACGTGTGCTCTTCCGATC*T** |
| **Primer2_Index12** | **CAAGCAGAAGACGGCATACGAGATTACAAGGTGACTGGAGTTCAG**  **ACGTGTGCTCTTCCGATC*T** |
| **Primer2_Index23** | **CAAGCAGAAGACGGCATACGAGATCCACTCGTGACTGGAGTTCAG**  **ACGTGTGCTCTTCCGATC*T** |
| **RTqPCR primers** | |
| **GAPDH up** | **GAAGGTGAAGGTCGGAGTCA** |
| **GAPDH dn** | **TTGAGGTCAATGAAGGGGTC** |
| **B2M up** | **TAGCTGTGCTCGCGCTACT** |
| **B2M dn** | **TCTCTGCTGGATGACGTGAG** |
| **PGK1 up** | **CAAGCTGGACGTTAAAGGGA** |
| **PGK1 dn** | **CTTGGGACAGCAGCCTTAAT** |
| **TFII-IcDNA up** | **CAAGGAAGCCACCATTCTTC** |
| **TFII-IcDNA dn** | **TAGCTCATTGGCCTTTGGTC** |
| **DNMT1cDNA up** | **AAG CAA GAA GTG AAG CCC GT** |
| **DNMT1cDNA dn** | **CTT AGC CTC TCC ATC GGA CT** |
| **CD81cDNA up** | **ACT CTC TGC CTT CAT GCA CC** |
| **CD81cDNA dn** | **CCT GGT AGC ATG CCT GAT GT** |
| **GATA1cDNA1 up** | **AAA CGG GCA GGT ACT CAG TG** |
| **GATA1cDNA1 dn** | **CGG TTC ACC TGG TGT AGC TT** |
| **MLL2cDNA up** | **GCC AGC AAA GCC TCT TCA** |
| **MLL2cDNA dn** | **TCT GAA GAA ACG GCT GGG TC** |
| **EFR3AcDNA up** | **GCA GAA AGG TTG AGC AGG GA** |
| **EFR3AcDNA dn** | **CTT TGG TTC CCC CGA TTC CA** |
| **ZAP70cDNA up** | **CACATTGCTCACAGGGATCT** |
| **ZAP70cDNA dn** | **CTGGACAACCCCTACATCGT** |
| **OSMcDNA up** | **CCC AGA CTT CCT CCT TTC CG** |
| **OSMcDNA dn** | **TGA GGT CAC CCA GAC ATC CA** |
| **ATF3Junc1 up** | **GGATTTTCAGCACCTTGCCC** |
| **ATF3Junc1 dn** | **TTGACAAAGGGCGTCAGGTT** |
| **ATF3Junc2 up** | **CTCGGGGTGTCCATCACAAA** |
| **ATF3Junc2 dn** | **GGCACTCCGTCTTCTCCTTC** |
| **ATF3Junc3 up** | **AGTGCCTGCAGAAAGAGTCG** |
| **ATF3Junc3 dn** | **AATACACGTGGGCCGATGAA** |
| **FAM71A cDNA up** | **CAACCGTGTCCGTATGGTGA** |
| **UBB up** | **TGAGCTTGTTTGTGTCCCTG** |
| **UBB dn** | **CGGTAAGGGTTTTCACGAAG** |
| **FAM71A cDNA dn** | **AAGTTCTTTGCTGCCTTGCG** |
| **ChIPqPCR primers** | |
| **HS2-3 up** | **CCTTGGTCAAGCTGCAACTT** |
| **HS2-3 dn** | **AAGACGGAGCCAATGGGTTA** |
| **ATF3Tpdn up** | **TGGGCCAACTAGGTCCATTTA** |
| **ATF3Tpdn dn** | **GGTTTGGAGATCAGTCCACGA** |
| **ATF3Pol2dn up** | **TTCTTCTAAGCCACCGCTGC** |
| **ATF3Pol2dn dn** | **CCGTCACCAGGAACCTTTCG** |
| **GATA1 ChIP up** | **GAG TGA GCC AGT CAG GGA AG** |
| **GATA1 ChIP dn** | **GCT GGG AGT GGG CAG ATA AG** |
| **DNMT1 ChIP up** | **AGG GTT TGT GAG AGC CCT TG** |
| **DNMT1 ChIP dn** | **ACT GTG AGA TTC TTG GTA CTA GC** |
| **EFR3A ChIP up** | **TGA GCA GAG CCA GGA AGT GT** |
| **EFR3A ChIP dn** | **GCC ATC TCC GGA AGG CTA A** |
| **OSM ChIP up** | **CACCTCACACCACTTCCCTG** |
| **OSM ChIP dn** | **GTCCACTGAGAACCTGGCAT** |
| **ATF3Pol2p1b up** | **GAAGGAACGAGGAGAGCCAC** |
| **ATF3Pol2p1b dn** | **GGTTGCTAATTTTAGCTCCGGC** |
| **ATF3Tp up** | **CTGCAGACTGAGAGCCCATC** |
| **ATF3Tp dn** | **CTTGGTTCAGGCTCAGAGGG** |
| **CD81-F** | **TCAACTCCTTCAGGAAGCCC** |
| **CD81-R** | **CGGGAGAACAACCCATTCCT** |
| **ZAP70-F** | **TTGAGCCACTTGTAGGCGAG** |
| **ZAP70-R** | **GTCAGAGAGGCCAAGCAACT** |
| **DNMT1upF** | **ACAGCAAAACACAGGCAACA** |
| **DNMT1upR** | **AGGGATCACAAGCATGAACC** |
| **DNMT1dnF** | **GTTCCTCTGACTCACGCACA** |
| **DNMT1dnR** | **AGCAGTTCATGCCTCTGTCC** |
| **EFR3AupF** | **TCTCCTCCCAGTACTGCACA** |
| **EFR3AupR** | **CCCCTGGGGACTTGTTGAAA** |
| **EFR3AdnF** | **ATGGGCCATGGTGGAGTTTAC** |
| **EFR3AdnR** | **ACACTGGTACCCCCAATCAA** |

**Note:** The * stands for phosphorothioate bond.

**Figure S1: Boxplot depicting the distribution of TFII-I peak distances around TSSs of expressed (green) and silent (red) target genes.** P values refer to the difference between TFII-I peak distances assigned to expressed and silent genes mapping either upstream (left, p=7e-4) or downstream (right, p=6e-4) of the genes’ TSS (Wilcoxon rank sum test).

**Figure S2. Binding of TFII-I at 8 gene loci that revealed a peak of the bio-tagged protein, and occupancy of TFII-I, , TAF15, and Elongin A (TCEB3) at three different regions of the DNMT1 and EFR3A gene loci. (A)** TFII-I occupancy at 8 different gene loci in K562 cells. K562 cells were subjected to ChIP and the purified DNA was analyzed by qPCR using primers specific for the genes as indicated. The data represent the results from two independent ezxperiments with the qPCR performed in triplicate. The relative enrichment of TFII-I at all genes compared to IgG control was found to be statistically significant (p<0.05). (B) TFII-I, TAF15, Elongin A (TCEB3), and Pol II occupancy at three different regions at the DNMT1 and EFR3A gene loci. K562 cells were subjected to ChIP and the purified DNA was analyzed with primers specific for the TFII-I peak (DNMT1 and EFR3A), or regions 1 Kb upstream (DNMT1up, EFR3Aup) or 1 Kb downstream (DNMT1dn, EFR3Adn) of the TFII-I peak. The data represent the results from two (EFR3A) or three (DNMT1) independent experiments with the PCR performed in triplicate. The data showing reduced binding of TFII-I, Elongin A and Pol II at the upstream and downstream regions of the DNMT1 and EFR3A genes were statistically significant (p<0.05) as was the reduced binding to TAF15 with the upstream and downstream regions of the DNMT1 gene.

**Figure S3. TFII-I and Pol II binding peaks at stress responsive genes.**

**Figure S4. Transcription factor binding and histone modifications associated with the TFII-I binding peak 5 kb upstream of the *ATF3* P1 promoter.**

**Figure S5. Interactions between the upstream TFII-I/Pol II binding peak and the promoter P2 in the ATF3 gene locus.** The Pol II ChIA-Pet interaction data retrieved from the ENCODE Project reflect the analysis of two different K562 cell clones (depicted on the left as 1 and 2). The thick black bars depict regions of interactions

**Figure S6 Protein/chromatin interactions and RNA levels in the ATF3 gene locus in the presence or absence of histidinol (HisOH) or in the presence or absence of siRNA directed against TFII-I, as described in the legend to Figs. 9C and D.**
